# Integrating serological and drainage fluid indicators: developing two predictive models for early detection of postoperative intra-abdominal infections in gastrointestinal tumor patients

**DOI:** 10.3389/fonc.2025.1566954

**Published:** 2025-04-22

**Authors:** Junfeng Zhou, Lin Lin, Cankun He, Ziyi Wang, Yuping Zhan, Sida Sun, Qingliang He

**Affiliations:** ^1^ Department of Gastrointestinal Surgery, The First Affiliated Hospital of Fujian Medical University, Fuzhou, China; ^2^ Nursing Department, The First Affiliated Hospital of Fujian Medical University, Fuzhou, China; ^3^ Department of General Surgery, Huian County Hospital, Hui’an, China; ^4^ Emergency Department, Zigong First People’s Hospital, Zigong, China; ^5^ Department of Infectious diseases, The First Affiliated Hospital of Fujian Medical University, Fuzhou, China; ^6^ Department of Infectious diseases, National Regional Medical Center, Binhai Campus of the First Affiliated Hospital, Fujian Medical University, Fuzhou, China; ^7^ Department of Gastrointestinal Surgery, National Regional Medical Center, Binhai Campus of the First Affiliated Hospital, Fujian Medical University, Fuzhou, China

**Keywords:** gastrointestinal tumor, postoperative intra-abdominal infection, drainage fluid, nomogram, surgery

## Abstract

**Purpose:**

This study aimed to investigate the influencing factors of postoperative intra-abdominal infection (PIAI) in gastrointestinal cancer patients by combining biomarkers in serum and drainage fluid (DF). It also intended to construct the predictive models and explore their predictive value for PIAI, offering clinical guidance.

**Methods:**

383 patients from Institution A formed the development cohort, and 77 patients from Institution B formed the validation cohort. Independent predictors of PIAI were identified using LASSO and logistic regression analysis based on biomarkers in serum and DF, and the corresponding nomograms were constructed. The nomograms were evaluated for their performance using the calibration curve, area under the curve (AUC), decision curve analysis (DCA), and clinical impact curve (CIC).

**Results:**

The prevalence of PIAI was 15.9% in the development cohort and 24.7% in the validation cohort. There were 5 indicators included in the nomogram on postoperative day (POD) 1, and 4 indicators on POD 3, including DF lactate dehydrogenase and C-reactive protein. The AUC values of the models in the development and validation cohorts were 0.731 and 0.958 on POD 1, and 0.834 and 0.951 on POD 3, respectively. The calibration curve, DCA, and CIC demonstrated the favorable clinical applicability of the models.

**Conclusions:**

Two nomogram models including serum and DF biomarkers on POD 1 and POD 3 were developed and validated. These models can identify patients at risk of PIAI and have promise for clinical application.

## 1 Introduction

Surgery remains the mainstay of treatment for gastrointestinal tumors; however, postoperative complications cast a significant shadow over patients, prolonging their hospital stay, escalating medical costs, and potentially compromising their long-term survival prospects (1–4). Postoperative intra-abdominal infection (PIAI), which includes anastomotic leaks, is one of the most common and severe complications after gastrointestinal tumor surgery, with an incidence rate of 2.8%-30% and a related mortality as high as 20% (5–7). Identifying PIAI is still challenging for clinicians, partly due to its heterogeneous clinical presentation, such as varied symptoms of fever, abdominal pain, and abnormal white blood cell (WBC) counts, which makes differential diagnosis difficult in the context of the systemic physiologic inflammatory response due to surgical stress (8, 9). Therefore, early identification and appropriate interventional measures are crucial, as they can mitigate patient complications and enhance clinical prognoses, especially in the era of Enhanced Recovery After Surgery (ERAS) programs (10–12).

Recently, there has been increasing interest in developing tools for the early detection of PIAI, including biomarkers in serum and drainage fluid (DF). Previous studies have shown that elevated levels of procalcitonin (PCT), a biomarker released in response to bacterial infection, and C-reactive protein (CRP), an acute-phase protein whose production is upregulated during inflammation, on the third day after surgery are associated with PIAI (10, 13). Additionally, regular measurement of albumin (ALB) levels early after surgery may help detect postoperative infectious complications, as decreased ALB levels can indicate a systemic inflammatory response and compromised nutritional status often associated with infections (14). However, all the previously mentioned indicators are serologic. Inflammatory markers within DF, such as lactate dehydrogenase (LDH), have been revealed to have a significant correlation with PIAI (15). We assume that DF markers are valuable because they can reflect local inflammatory changes within the abdominal cavity, which may precede systemic manifestations. Additionally, we believe that DF markers can provide an effective approach for early detection and serve as a potent complement to serum markers, thereby expanding the toolkit for PIAI diagnosis.

In this study, we did not limit ourselves to serological or DF indicators; instead, we combined both types and constructed two predictive models based on the identification of independent risk factors. We expect that our findings will enable clinicians to identify PIAI in its initial stages, allowing for timely intervention and treatment, which could lead to a reduction in patient complications and postoperative mortality.

## 2 Materials and methods

### 2.1 Study design and population

This was a two-center retrospective study of 556 consecutive patients who underwent gastric or colorectal cancer surgery between April 2023 and August 2024. Of these, 453 patients from Institution A were included in the development cohort, and 103 patients from Institution B were included in the validation cohort. The data from these two cohorts were entirely independent of each other, and efforts were made to ensure consistency in the surgical procedures and data collection protocols across both centers. According to the diagnostic criteria for intra-abdominal infections by the Centers for Disease Control and Prevention (16), the patients were divided into the PIAI group and the non-PIAI group, based on whether they developed PIAI after surgery. The study was performed in accordance with the tenets of the Declaration of Helsinki and was approved by the ethics committee of First Affiliated Hospital of Fujian Medical University (No. MTCA, ECFAH of FMU [2015] 084-2). Moreover, the requirement for written informed consent was waived due to the retrospective nature of our study.

The inclusion criteria for patients were as follows: (1) patients with gastrointestinal tumor undergoing surgical treatment; (2) postoperative collection of abdominal DF for examination; (3) postoperative pathological diagnosis of gastrointestinal tumors. The exclusion criteria were as follows: (1) patients with preoperative abdominal infection; (2) no postoperative collection of abdominal DF for examination; (3) postoperative pathology of patients with benign lesions; (4) non-radical resection; (5) patients with over 20% missing data. Both the development and validation cohorts adhered to the same inclusion and exclusion criteria.

### 2.2 Data collection

After disposing of the DF from the previous day, the same clinician collected the abdominal DF and immediately sent it for laboratory analysis to prevent glycolysis in the sample from affecting the glucose values in the results. The collection of abdominal DF was performed on postoperative day (POD) 1 and 3. Clinicopathologic data were collected, including gender, age, body mass index (BMI), nutritional risk screening (NRS) 2002 score (17), American Society of Anesthesiologists (ASA) physical status classification (18), history of neoadjuvant chemotherapy (NACT), tumor location, and Tumor-Node-Metastasis (TNM) classification (19). Additionally, preoperative WBC, hemoglobin (HB), and serum ALB levels were collected. On POD 1 and POD 3, serum biomarkers, such as WBC, neutrophil ratio (NEUT%), HB, CRP, PCT, interleukin-6 (IL-6), ALB, and DF biomarkers, including WBC, mononuclear cells, multinucleated cells, total protein (TP), LDH, amylase, and glucose (GLU), were gathered.

### 2.3 Statistical analysis

Statistical analyses were performed using SPSS statistical software v.22.0 (SPSS Inc., Armonk, NY, USA) and R Language v.4.0.3 (R Foundation for Statistical Computing, Vienna, Austria). A two-sided *P*-value of less than 0.05 was considered statistically significant. Normally distributed continuous variables were recorded as mean ± standard deviation and analyzed using Student’s t-test, while non-normally distributed continuous variables were recorded as median (25th and 75th percentiles) and analyzed using non-parametric tests. Categorical data were expressed as frequencies (%) and compared using chi-squared or Fisher’s exact test. Based on the normal range of indicators from these two institutions, preoperative serum indicators were transformed into binary variables, including WBC (0 = “WBC < 10 × 10^9^/L”, 1 = “WBC ≥ 10 × 10^9^/L”), albumin (0 = “ALB < 40 g/L”, 1 = “ALB ≥ 40 g/L”), and hemoglobin (0 = “HB ≥ 110 g/L”, 1 = “90 g/L ≤ HB < 110 g/L”, 2 = “60 g/L ≤ HB < 90 g/L”, 3 = “HB < 60 g/L”) for subsequent analysis (20–22). The collinearity test was checked by running a collinearity diagnostic with the variance inflation factor (VIF) statistic, and selected variables were excluded if the VIF was greater than 10 until no collinearity existed (23, 24). The least absolute shrinkage and selection operator (LASSO) regression analysis was conducted for data dimensionality reduction and screening. Furthermore, the algorithm’s iterations were set to 1,000 to ensure precision, and the cv.glmnet function was employed to perform 10-fold cross-validation to reduce the likelihood of overfitting (25). Predictors of PIAI were examined using univariate and multivariate logistic regression. Based on the results of the multivariable analysis, the nomograms were created to calculate the estimated probability of PIAI. The performance of the models was evaluated using several metrics, including the calibration curve, receiver operating characteristic (ROC) curve, decision curve analysis (DCA), and clinical impact curve (CIC), in both the development and validation cohorts.

## 3 Results

### 3.1 Study population and baseline characteristics

**Figure 1:**
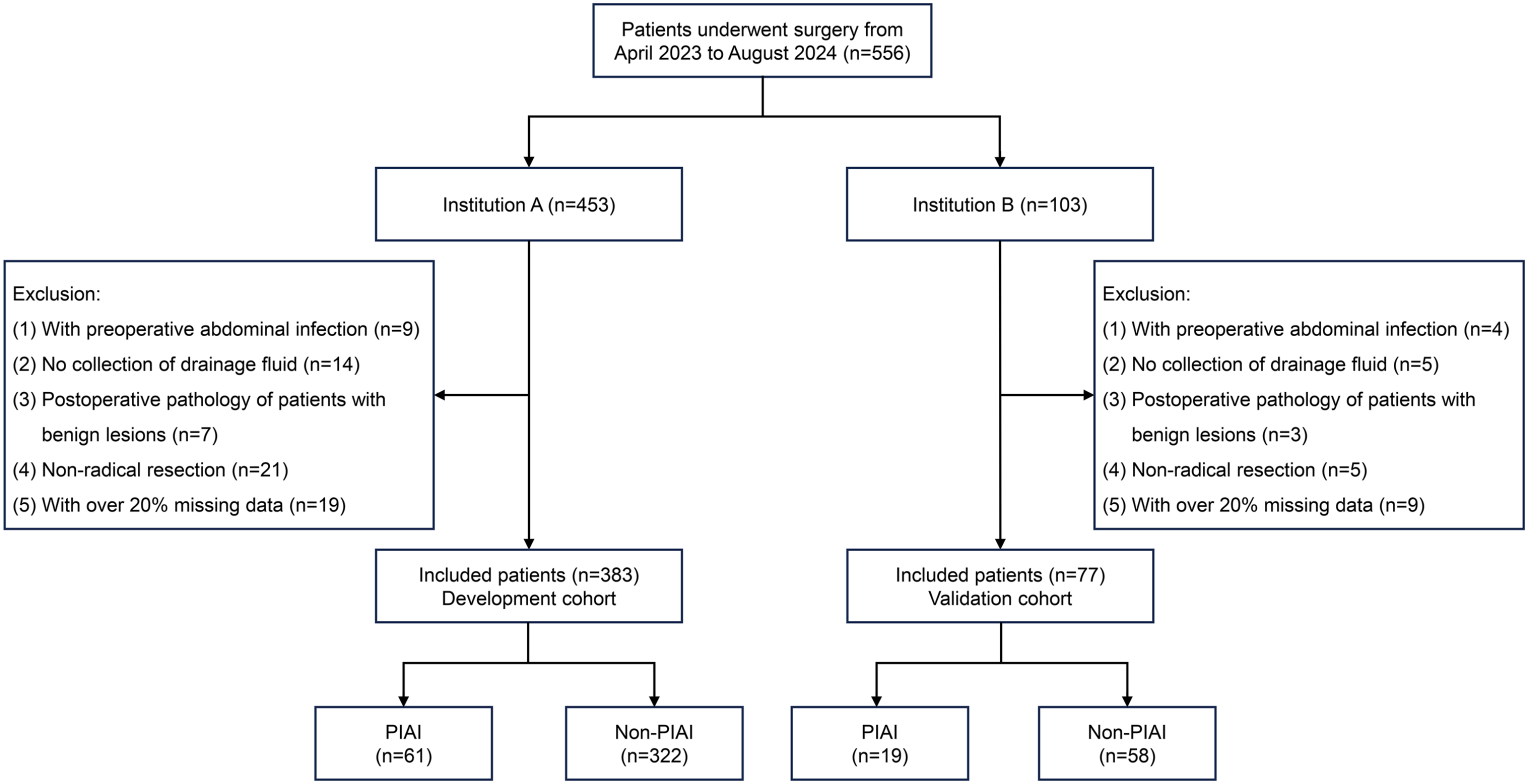
Flow diagram of patients included in this study. PIAI, postoperative intra-abdominal infection.

A total of 556 patients were reviewed (Institution A, n=453; Institution B, n=103). Of these, 460 were included, and 96 were excluded for the following reasons: preoperative abdominal infection (n=13), lack of postoperative abdominal DF collection for examination (n=19), postoperative pathology indicating benign lesions (n=10), non-radical resection (n=26), and more than 20% missing data (n=28). **Figure 1** illustrates the inclusion and exclusion process for the development and validation cohorts. Meanwhile, the characteristics of patients and PIAI-related information in both cohorts are clearly presented in **Table 1**. Specifically, for the development cohort, the patients (258 males and 125 females) had a median age of 61 years, and the prevalence of PIAI was 15.9%. For the validation cohort, the patients (49 males and 28 females) had a median age of 59 years, and the prevalence of PIAI was 24.7%. Furthermore, the mean time for the occurrence of PIAI was 5.3 days for all included patients.

**Table 1:** Characteristics of patients and PIAI-related information in development and validation cohorts.

Characteristic	Total	Development cohort (n=383)	Validation cohort (n=77)
Mean age (years)	60 (55, 69)	61 (56, 69)	59 (54, 68)
Gender (%)			
Male	307 (66.7%)	258 (67.4%)	49 (63.6%)
Female	153 (33.3%)	125 (32.6%)	28 (36.4%)
BMI (kg/m^2^)			
≤18.5	39 (8.5%)	32 (8.4%)	7 (9.1%)
≤24	289 (62.8%)	233 (60.8%)	56 (72.7%)
≤28	98 (21.3%)	87 (22.7%)	11 (14.3%)
>28	34 (7.4%)	31 (8.1%)	3 (3.9%)
NRS 2002 score			
<3	275 (59.8%)	224 (58.5%)	51 (66.2%)
≥3	185 (40.2%)	159 (41.5%)	26 (33.8%)
ASA grade (%)			
<3	406 (88.3%)	340 (88.8%)	66 (85.7%)
≥3	54 (11.7%)	43 (11.2%)	11 (14.3%)
History of NACT			
Yes	435 (94.6%)	370 (96.6%)	65 (84.4%)
No	25 (5.4%)	13 (3.4%)	12 (15.6%)
Tumor location			
Stomach	267 (58%)	227 (59.3%)	40 (51.9%)
Colorectum	193 (42%)	156 (40.7%)	37 (48.1%)
Preoperative serum albumin (g/L)			
<40	233 (50.7%)	193 (50.4%)	40 (51.9%)
≥40	227 (49.3%)	190 (49.6%)	37 (48.1%)
Preoperative hemoglobin (g/L)			
≥110	331 (72.0%)	267 (69.7%)	64 (83.1%)
≥90	81 (17.6%)	72 (18.8%)	9 (11.7%)
<90	48 (10.4%)	44 (11.5%)	4 (5.2%)
Preoperative WBC (10^9^/L)			
<10	421 (91.5%)	352 (91.9%)	69 (89.6%)
≥10	39 (8.5%)	31 (8.1%)	8 (10.4%)
Surgical duration (hours)	4.1 (3.2, 4.9)	4.0 (3.2, 4.8)	4.5 (3.7, 5.6)
POD1 WBC (10^9^/L)	10.78 (8.68, 13.15)	11.27 (8.99, 13.04)	8.68 (7.02, 16.16)
POD1 hemoglobin (g/L)	118.0 (104.0, 131.0)	120.0 (105.5, 130.5)	121.1 (90.8, 135.8)
POD1 NEUT%	84.6 (81.1, 89.2)	84.7 (81.5, 89.2)	82.8 (75.0, 89.2)
POD1 platelet (10^9^/L)	229 (173, 294)	228 (173, 283)	258 (168, 334)
POD1 CRP (mg/L)	23.50 (17.39, 39.50)	21.29 (14.74, 35.40)	37.83 (19.89, 52.49)
POD1 PCT (ng/ml)	0.32 (0.11, 0.79)	0.24 (0.07, 0.65)	0.71 (0.43, 1.88)
POD1 IL6 (pg/ml)	31.83 (20.97, 61.16)	30.41 (18.94, 46.23)	84.69 (40.53, 150.30)
POD1 serum albumin (g/L)	33.60 (31.40, 35.80)	33.70 (31.50, 35.80)	32.84 (29.55, 35.85)
POD1 DF WBC (10^6^/L)	8993 (3326, 18769)	7521 (3261, 18354)	13627 (8482, 23151)
POD1 DF monocyte (10^6^/L)	690 (253, 1789)	535 (243, 1040)	4814 (1932, 6934)
POD1 DF polykaryocyte (10^6^/L)	6771 (2300, 15824)	6677 (2219, 15874)	7883 (5152, 14658)
POD1 DF TP (g/L)	37.89 (34.80, 43.80)	37.60 (34.60, 43.00)	39.51 (36.06, 44.84)
POD1 DF LDH (U/L)	1078 (768, 1468)	996 (697, 1464)	1222 (882, 1575)
POD1 DF amylase (U/L)	264 (52, 464)	212 (45, 565)	321 (212, 392)
POD1 DF glucose (mmol/L)	5.42 (4.17, 7.03)	5.42 (4.01, 7.46)	5.19 (4.51, 6.29)
POD3 WBC (10^9^/L)	7.62 (6.54, 9.47)	7.59 (6.44, 9.41)	8.27 (6.69, 10.05)
POD3 hemoglobin (g/L)	116 (104, 131)	115.0 (104.0, 132.0)	119.1 (108.5, 128.9)
POD3 NEUT%	78.45 (71.60, 82.58)	78.4 (71.6, 81.8)	79.7 (72.2, 88.0)
POD3 platelet (10^9^/L)	212 (163, 268)	204 (163, 263)	267 (187, 308)
POD3 CRP (mg/L)	49.71 (33.62, 86.57)	42.09 (28.67, 86.52)	73.87 (52.13, 97.87)
POD3 PCT (ng/ml)	0.30 (0.10, 0.74)	0.23 (0.09, 0.55)	0.98 (0.56, 1.72)
POD3 IL-6 (pg/ml)	22.72 (13.27, 35.54)	21.46 (13.67, 35.54)	25.95 (14.01, 33.29)
POD3 serum albumin (g/L)	33.00 (30.70, 35.23)	33.00 (30.75, 35.30)	32.83 (30.34, 34.46)
POD3 DF WBC (10^6^/L)	8271 (3590, 15761)	5971 (3129, 18205)	10389 (7896, 14132)
POD3 DF monocyte (10^6^/L)	1217 (495, 2584)	950 (471, 1830)	2987 (2169, 4669)
POD3 DF polykaryocyte (10^6^/L)	6168 (2964, 14170)	5722 (2378, 16718)	7154 (4949, 10144)
POD3 DF TP (g/L)	38.30 (34.88, 42.50)	37.80 (34.45, 42.50)	39.71 (37.60, 42.36)
POD3 DF LDH (U/L)	890 (613, 1267)	821 (594, 1280)	1045 (855, 1207)
POD3 DF amylase (U/L)	185 (76, 301)	134 (68, 351)	246 (197, 270)
POD3 DF glucose (mmol/L)	4.41 (3.44, 4.13)	4.66 (3.37, 5.76)	4.06 (3.62, 4.51)
TNM stage, n (%)			
<III	325 (70.7%)	274 (71.5%)	51 (66.2%)
III	135 (29.3%)	109 (28.5%)	26 (33.8%)
PIAI			
Yes	80 (17.4%)	61 (15.9%)	19 (24.7%)
No	380 (82.6%)	322 (84.1%)	58 (75.3%)
Time for occurrence of PIAI (days)	5.3 (4.6, 6.3)	5.4 (4.6, 6.3)	4.8 (4.2, 5.9)

BMI, body mass index; NRS, nutrition risk screening; ASA, American Society of Anesthesiologists; NACT, neoadjuvant chemotherapy; WBC, white blood cell; POD, postoperative days; NEUT%, neutrophil ratio; CRP, C-reactive protein; PCT, procalcitonin; IL-6, interleukin-6; DF, drainage fluid; TP, total protein; LDH, lactate dehydrogenase; TNM, Tumor-Node-Metastasis.

### 3.2 Data dimensionality reduction and variable selection

**Figure 2:**
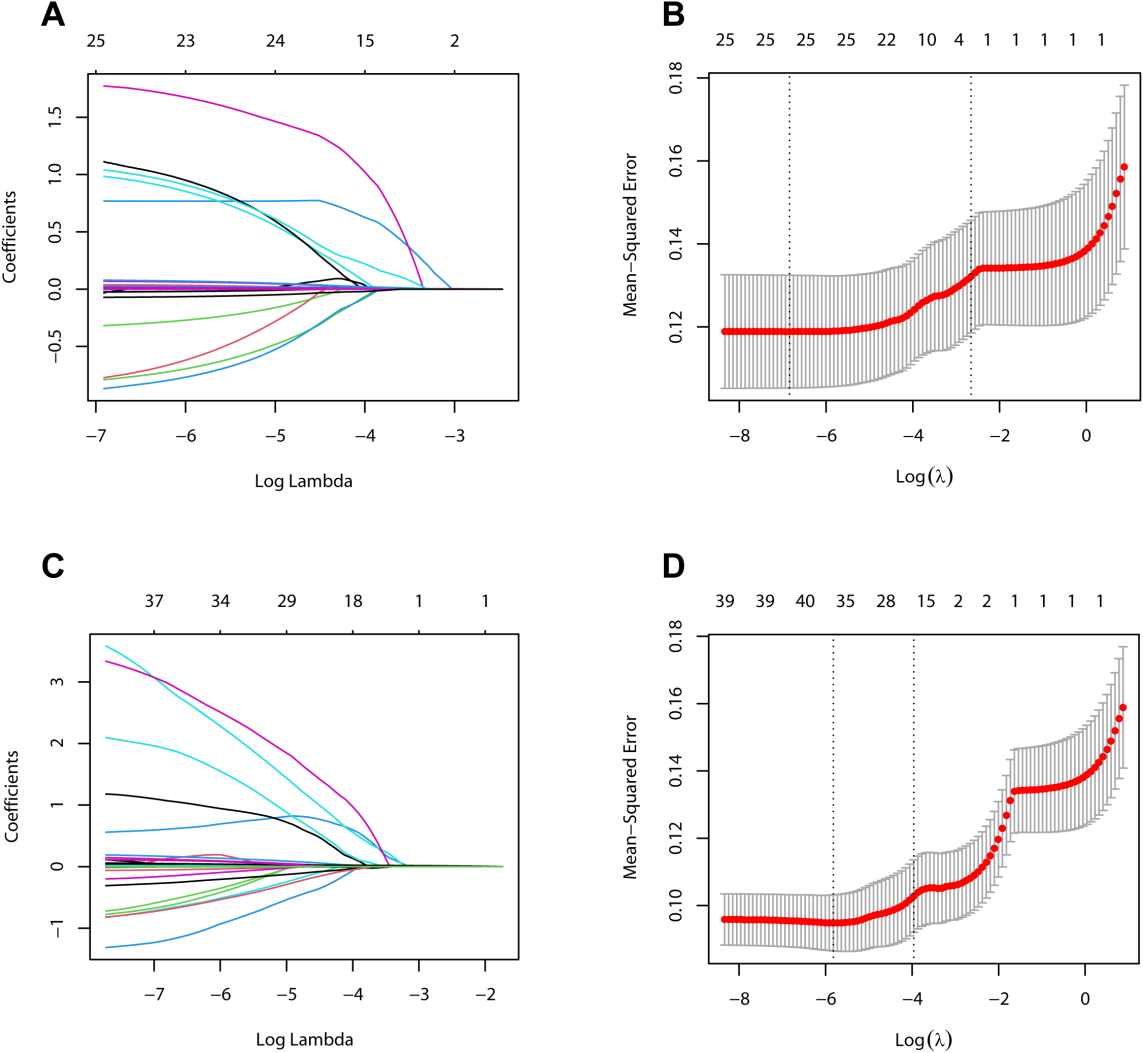
Variables selection using the LASSO regression method. **(A)** LASSO coefficient curves for 26 variables on postoperative day 1 in the development cohort. **(B)** Identification of the optimal penalty coefficient (λ) in LASSO regression model on postoperative day 1 in the development cohort. **(C)** LASSO coefficient curves for 40 variables on postoperative day 3 in the development cohort. **(D)** Identification of the optimal penalty coefficient (λ) in LASSO regression model on postoperative day 3 in the development cohort.

A total of 26 variables were analyzed in the development cohort on POD 1, including preoperative clinical pathological data, and serum and drainage biomarkers of POD 1. Initially, the DF WBC on POD 1 was excluded due to high collinearity with a VIF greater than 10. Consequently, the remaining 25 variables were incorporated into the LASSO regression analysis for 10-fold cross-validation (**Figures 2A, B**). Ultimately, all 25 variables were selected for subsequent logistic regression analysis.

In the analysis of variables in the development cohort on POD 3, the variables analyzed on POD 1 were included, along with serum and drainage indicators on POD 3. After collinearity analysis, the DF WBC on POD 1 was excluded, and the remaining 40 variables were incorporated into the LASSO regression analysis for 10-fold cross-validation (**Figures 2C, D**). Subsequently, NEUT% and platelet counts on POD 1, the platelet counts on POD 3, and the WBC and monocyte counts in the DF on POD 3 were excluded owing to their relatively minor contributions to the predictive power of the model. Ultimately, the remaining 35 variables were selected for subsequent logistic regression analysis.

### 3.3 Logistic regression analysis and construction of the nomograms

**Figure 3:**
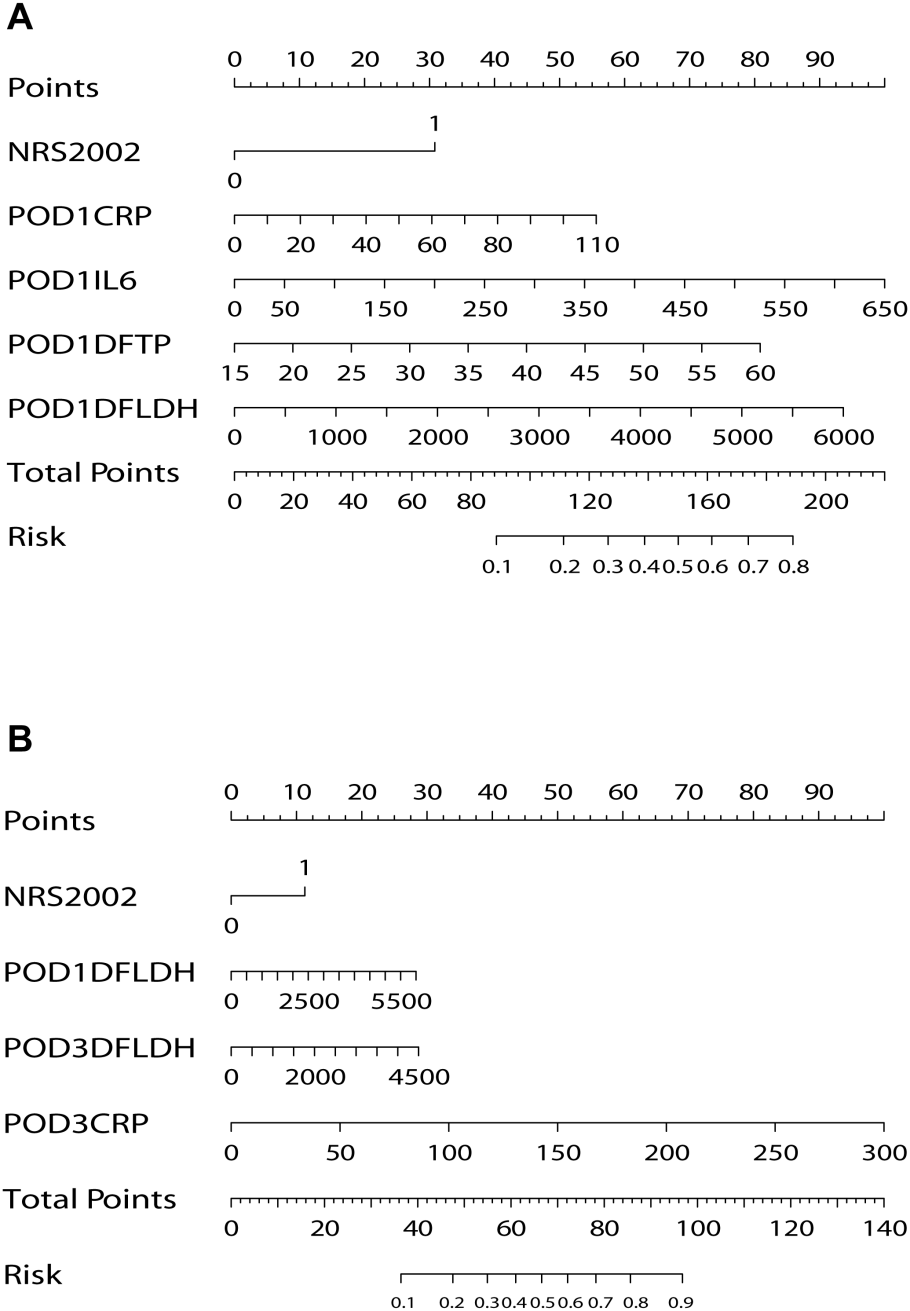
Nomograms for prediction of postoperative intra-abdominal infection. **(A)** Nomogram on postoperative day 1. **(B)** Nomogram on postoperative day 3. NRS, nutritional risk screening; POD, postoperative day; CRP, C-reactive protein; IL-6, interleukin-6; DF, drainage fluid; TP, total protein; LDH, lactate dehydrogenase.

We performed a univariate logistic regression analysis on the 25 variables collected on POD 1 (**Table 2**). Then, we selected 6 variables with *P*-values less than 0.05 for multivariate analysis. These variables included NRS2002, POD1 CRP, POD1 PCT, POD1 IL-6, POD1 DF TP, and POD1 DF LDH. The results of multivariate analysis revealed that NRS2002 (OR = 0.377; 95% CI: 0.187-0.760; *P* = 0.006), POD1 CRP (OR = 1.016; 95% CI: 1.001-1.031; *P* = 0.035), POD1 IL-6 (OR = 1.005; 95% CI: 1.002-1.009; *P* = 0.002), POD1 DF TP (OR = 1.064; 95% CI: 1.015-1.115; *P* = 0.010), and POD1 DF LDH (OR = 1.001; 95% CI: 0.993-1.010; *P* = 0.001) were all statistically significant and identified as independent predictors of PIAI. These 5 independent predictors were utilized to construct a predictive nomogram for PIAI (**Figure 3A**).

**Table 2:** Univariable and multivariable binary logistic regression analysis for risk factors of postoperative intra-abdominal infection in the development cohort on postoperative days 1.

Variables	Univariate Analysis		Multivariate Analysis	
	OR (95% CI)	*P* value	OR (95% CI)	*P* value
Gender, male vs female	0.766 (0.434-1.353)	0.358	–	–
Age, years	1.004 (0.978-1.031)	0.769	–	–
BMI, kg/m^2^	1.050 (0.726-1.519)	0.795	–	–
NRS2002, <3 vs ≥3	0.429 (0.246-0.750)	0.003	0.377 (0.187-0.760)	0.006
ASA, <3 vs ≥3	0.502 (0.237-1.060)	0.071	–	–
Tumor location, stomach vs colorectum	0.911 (0.524-1.586)	0.743	–	–
NACT, yes vs no	0.410 (0.122-1.376)	0.149	–	–
Preoperative WBC, <10 vs ≥10, 10^9^/L	0.543 (0.160-1.846)	0.328	–	–
Preoperative serum albumin, <40 vs ≥40, g/L	0.716 (0.412-1.244)	0.235	–	–
Preoperative hemoglobin, g/L	0.782 (0.506-1.208)	0.268	–	–
Surgical duration, hours	1.122 (0.920-1.368)	0.257	–	–
POD1 WBC, 10^9^/L	1.061 (0.987-1.141)	0.108	–	–
POD1 hemoglobin, g/L	1.008 (0.995-1.021)	0.220	–	–
POD1 NEUT%	1.049 (0.998-1.104)	0.060	–	–
POD1 platelet, 10^9^/L	1.001 (0.998-1.005)	0.524	–	–
POD1 CRP, mg/L	1.015 (1.002-1.029)	0.025	1.016 (1.001-1.031)	0.035
POD1 PCT, ng/ml	1.655 (1.024-2.674)	0.040	–	0.195
POD1 IL-6, pg/ml	1.006 (1.003-1.009)	<0.001	1.005 (1.002-1.009)	0.002
POD1 serum albumin, g/L	0.963 (0.891-1.040)	0.332	–	–
POD1 DF monocyte, 10^6^/L	1.001 (0.998-1.003)	0.984	–	–
POD1 DF polykaryocyte, 10^6^/L	1.003 (0.997-1.008)	0.721	–	–
POD1 DF TP, g/L	1.046 (1.005-1.089)	0.026	1.064 (1.015-1.115)	0.010
POD1 DF LDH, U/L	0.999 (0.994-1.008)	0.004	1.001 (0.993-1.010)	0.001
POD1 DF amylase, U/L	1.001 (0.998-1.005)	0.446	–	–
POD1 DF glucose, mmol/L	0.892 (0.794-1.002)	0.054	–	–

BMI, body mass index; NRS, nutrition risk screening; ASA, American Society of Anesthesiologists; NACT, neoadjuvant chemotherapy; WBC, white blood cell; POD, postoperative days; NEUT%, neutrophil ratio; CRP, C-reactive protein; PCT, procalcitonin; IL6, interleukin-6; DF, drainage fluid; TP, total protein; LDH, lactate dehydrogenase.

For the 40 variables measured on POD 3, a univariate logistic regression analysis was conducted (**Table 3**). Subsequently, 14 variables with *P*-values less than 0.05 were included in the multivariate analysis. Notably, among the significant results, NRS2002 (OR = 0.400, 95% CI: 0.178-0.900; *P* = 0.027), POD1 DF LDH (OR = 1.001, 95% CI: 0.997-1.006; *P* = 0.016), POD3 CRP (OR = 1.027, 95% CI: 1.017-1.037; *P* < 0.001), and POD3 DF LDH (OR = 1.002, 95% CI: 0.997-1.009; *P* = 0.038) were identified as independent predictors of PIAI. These 4 independent predictors were used to construct another predictive nomogram for PIAI (**Figure 3B**).

**Table 3:** Univariable and multivariable binary logistic regression analysis for risk factors of postoperative intra-abdominal infection in the development cohort on postoperative days 3.

Variables	Univariate Analysis		Multivariate Analysis	
	OR (95% CI)	*P* value	OR (95% CI)	*P* value
Gender, male vs female	0.766 (0.434-1.353)	0.358	–	–
Age, years	1.004 (0.978-1.031)	0.769	–	–
BMI, kg/m^2^	1.050 (0.726-1.519)	0.795	–	–
NRS2002, <3 vs ≥3	0.429 (0.246-0.750)	0.003	0.400 (0.178-0.900)	0.027
ASA, <3 vs ≥3	0.502 (0.237-1.060)	0.071	–	–
Tumor location, stomach vs colorectum	0.911 (0.524-1.586)	0.743	–	–
NACT, yes vs no	0.410 (0.122-1.376)	0.149	–	–
Preoperative WBC, <10 vs ≥10, 10^9^/L	0.543 (0.160-1.846)	0.328	–	–
Preoperative serum albumin, <40 vs ≥40, g/L	0.716 (0.412-1.244)	0.235	–	–
Preoperative hemoglobin, g/L	0.782 (0.506-1.208)	0.268	–	–
Surgical duration, hours	1.122 (0.920-1.368)	0.257	–	–
POD1 WBC, 10^9^/L	1.061 (0.987-1.141)	0.108	–	–
POD1 hemoglobin, g/L	1.008 (0.995-1.021)	0.220	–	–
POD1 NEUT%	1.049 (0.998-1.104)	0.060	–	–
POD1 platelet, 10^9^/L	1.001 (0.998-1.005)	0.524	–	–
POD1 CRP, mg/L	1.015 (1.002-1.029)	0.025	–	0.066
POD1 PCT, ng/ml	1.655 (1.024-2.674)	0.040	–	0.178
POD1 IL-6, pg/ml	1.006 (1.003-1.009)	<0.001	–	0.814
POD1 serum albumin, g/L	0.963 (0.891-1.040)	0.332	–	–
POD1 DF monocyte, 10^6^/L	1.001 (0.998-1.003)	0.984	–	–
POD1 DF polykaryocyte, 10^6^/L	1.003 (0.997-1.008)	0.721	–	–
POD1 DF TB, g/L	1.046 (1.005-1.089)	0.026	–	0.124
POD1 DF LDH, U/L	0.999 (0.994-1.008)	0.004	1.001 (0.997-1.006)	0.016
POD1 DF glucose, mmol/L	0.892 (0.794-1.002)	0.054	–	
POD3 WBC, 10^9^/L	1.122 (1.010-1.247)	0.031	–	0.967
POD3 platelet, 10^9^/L	1.002 (0.998-1.005)	0.287	–	–
POD3 CRP, mg/L	1.026 (1.019-1.033)	<0.001	1.027 (1.017-1.037)	<0.001
POD3 PCT, ng/ml	1.157 (1.010-1.326)	0.036	–	0.452
POD3 IL-6, pg/ml	1.003 (1.001-1.004)	0.001	–	0.077
POD3 serum albumin, g/L	0.893 (0.818-0.975)	0.011	–	0.996
POD3 DF WBC, 10^6^/L	1.002 (0.998-1.005)	0.058	–	–
POD3 DF TP, g/L	1.057 (1.011-1.105)	0.015	–	0.881
POD3 DF LDH, U/L	0.997 (0.991-1.005)	0.046	1.002 (0.997-1.009)	0.038
POD3 DF amylase, U/L	1.001 (0.999-1.002)	0.308	–	–
POD3 DF glucose, mmol/L	0.826 (0.691-0.986)	0.034	–	0.055

BMI, body mass index; NRS, nutrition risk screening; ASA, American Society of Anesthesiologists; NACT, neoadjuvant chemotherapy; WBC, white blood cell; POD, postoperative days; NEUT%, neutrophil ratio; CRP, C-reactive protein; PCT, procalcitonin; IL6, interleukin-6; DF, drainage fluid; TP, total protein; LDH, lactate dehydrogenase.

### 3.4 Validation of the nomograms

**Figure 4:**
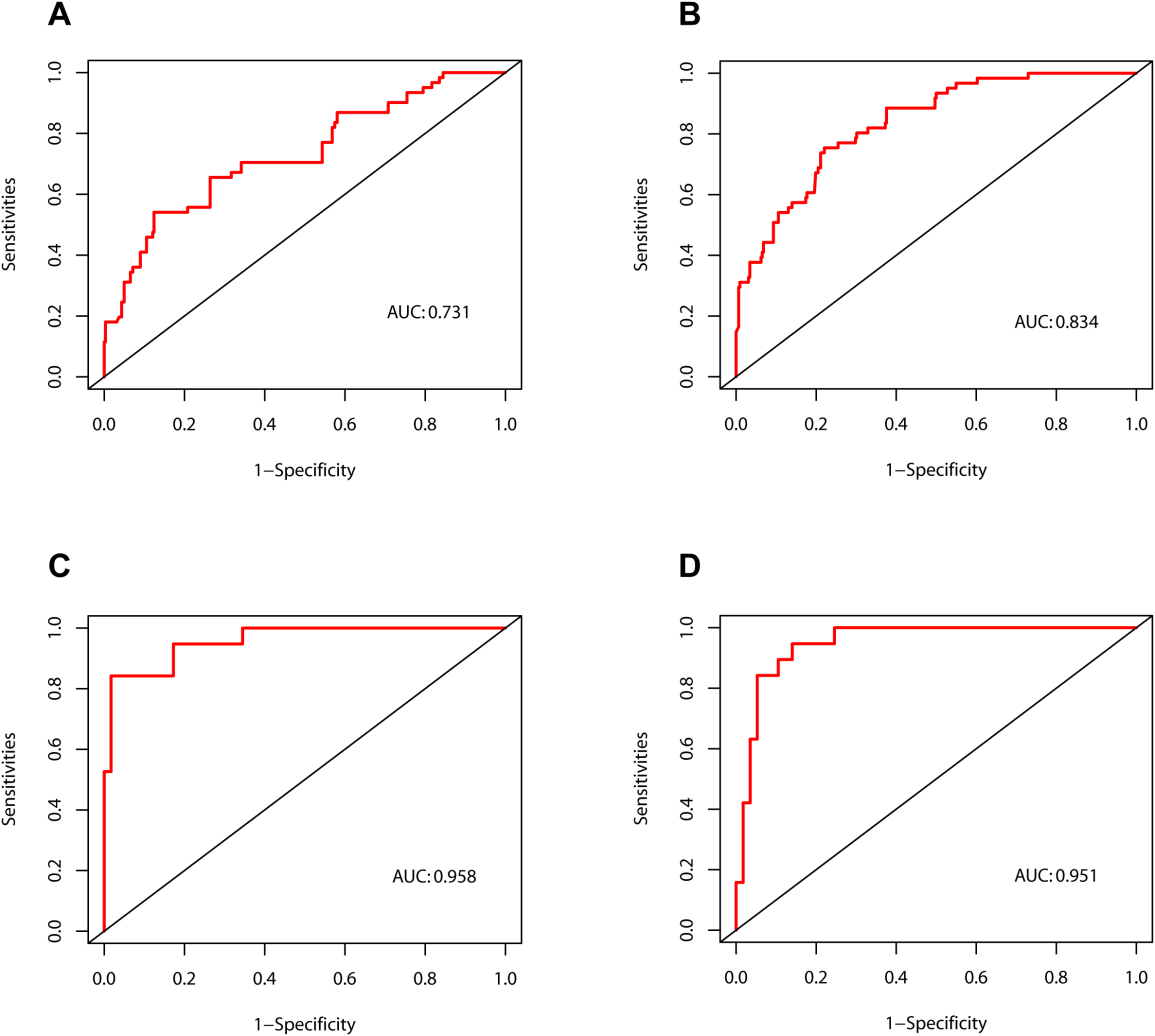
Calibration curves of the nomograms. **(A)** The development cohort on postoperative day 1. **(B)** The development cohort on postoperative day 3. **(C)** The validation cohort on postoperative day 1. **(D)** The validation cohort on postoperative day 3.

**Figure 5:**
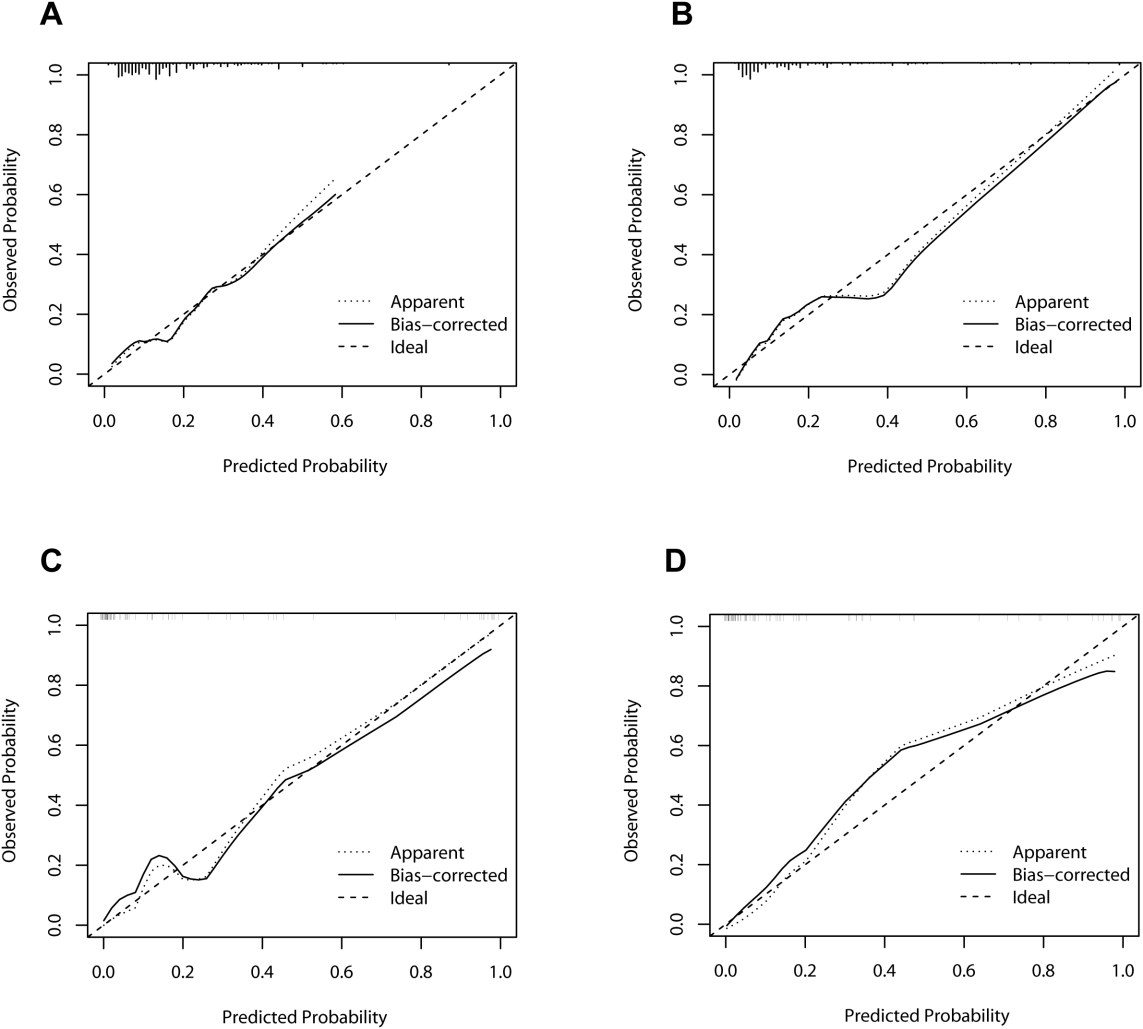
Receiver operating characteristic curves of the nomograms. **(A)** The development cohort on postoperative day 1. **(B)** The development cohort on postoperative day 3. **(C)** The validation cohort on postoperative day 1. **(D)** The validation cohort on postoperative day 3. AUC, area under curve.

As illustrated in **Figure 4**, the calibration curves for POD 1 and POD 3 in both the development and validation cohorts exhibited a high degree of correlation with the ideal diagonal line, thereby substantiating the efficacy of the nomograms. Additionally, the AUC values for POD 1 and POD 3 in the development cohort were 0.731 and 0.834, respectively (**Figures 5A, B**). In the validation cohort, these values were 0.958 and 0.951 (**Figures 5C, D**). These results indicated that the nomograms exhibited great performance.

**Figure 6:**
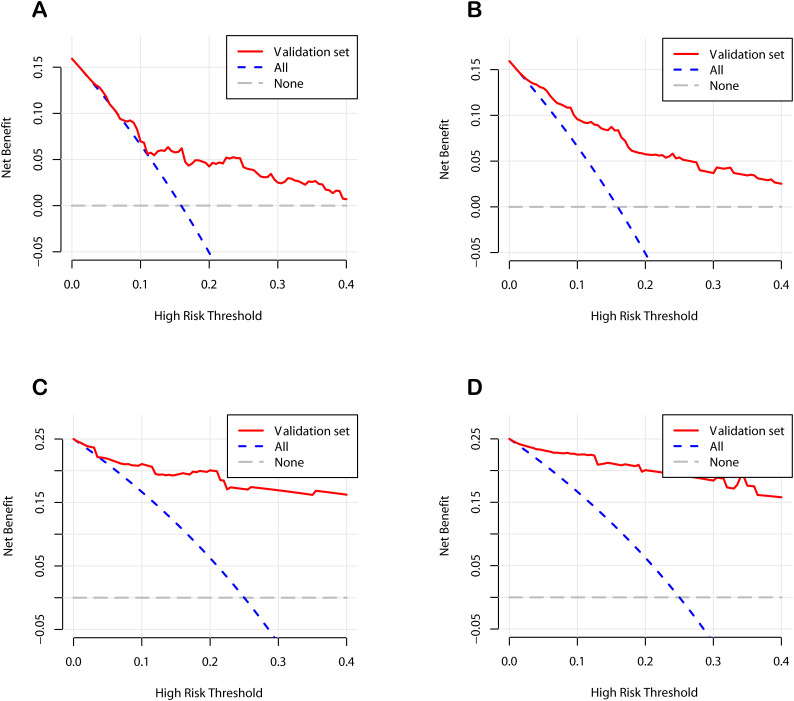
Decision curve analysis curves of the nomograms. **(A)** The development cohort on postoperative day 1. **(B)** The development cohort on postoperative day 3. **(C)** The validation cohort on postoperative day 1. **(D)** The validation cohort on postoperative day 3.

**Figure 7:**
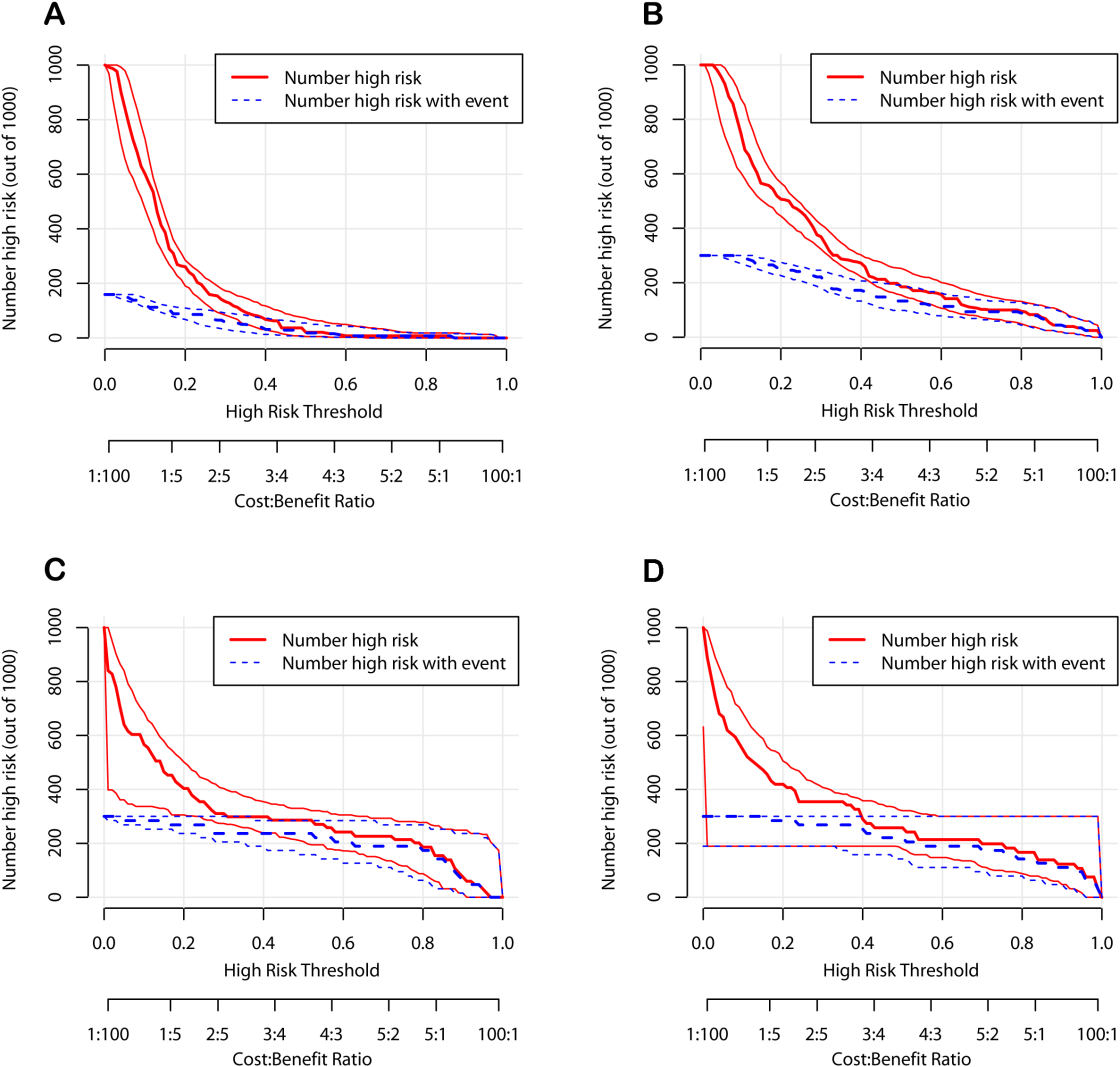
Clinical impact curves of the nomograms. **(A)** The development cohort on postoperative day 1. **(B)** The development cohort on postoperative day 3. **(C)** The validation cohort on postoperative day 1. **(D)** The validation cohort on postoperative day 3.

To further verify the clinical applicability of the nomogram models, we employed DCA and CIC. DCA assesses the net benefit of a prediction model by comparing it to “full intervention” and “no intervention” strategies. For POD 1 and POD 3 in both the development and validation cohorts, the DCA demonstrated that the nomograms exhibited superior net benefits compared to these strategies (**Figure 6**). Specifically, the cutoff value for the POD 1 model was set at 24.6%, and for the POD 3 model at 15.1%, based on the points of maximum net benefit on the DCA curves. CIC, derived from DCA, shows the relationship between the high-risk threshold and the number of true positives. It indicated that as the high-risk threshold increased, the number of true positives increased proportionally, suggesting favorable net clinical benefit (**Figure 7**). In both the development and validation cohorts, the nomograms consistently demonstrated good predictive performance. These results indicated that the nomograms exhibited considerable generalizability and clinical applicability.

## 4 Discussion

PIAI is a common but potentially life-threatening complication following gastrointestinal surgery, particularly in patients with gastrointestinal cancer who are already immunocompromised (26). It is associated with high postoperative mortality rates, prolonged hospital stays, and increased costs of treatment (3, 5, 27). Accordingly, the timely and appropriate initiation of treatment is essential. In our study, for early detection of PIAI, we integrated serological and DF indicators to identify the influencing factors, thereby developing the predictive models. This combined approach allows for a more comprehensive assessment, potentially enhancing the accuracy of our models in identifying patients at risk of PIAI. As a result, the nomograms were developed for PIAI on POD 1 and POD 3 with favorable predictive performance, indicating its potential generalization and clinical utility.

The early manifestations of PIAI are insidious and heterogeneous, presenting significant challenges for clinicians in early diagnosis (27). By the time a conclusive diagnosis is made, patients often progress to develop serious conditions, such as high fever, abdominal pain, and even sepsis (8). Consequently, the median time interval from surgery to the diagnosis of PIAI remains a subject of contention. Several studies have indicated that the median time to determine PIAI after colorectal surgery was 7 to 10 days postoperatively (28, 29). In contrast, within our study, the mean time to diagnose PIAI was as short as 5.3 days. This was achieved because the postoperative abdominal DF was immediately sent for examination. The inflammatory indicators in the abdominal DF are likely to have a sensitive correlation with PIAI (15). Therefore, analyzing these indicators in the abdominal DF serves as an effective approach for the early detection of PIAI. It also acts as a powerful complement to serum markers. Moreover, in our study, the overall prevalence of PIAI was 17.4%, which is consistent with a study investigating PIAI after gastrointestinal tumor surgery (13), but higher than the postoperative morbidity of gastric cancer patients after gastrectomy reported in other studies (30, 31). Our findings further confirm the high incidence and severity of PIAI following gastrointestinal tumor surgery, providing valuable reference for future research.

It was unsurprisingly found that an NRS2002 score ≥ 3 was an independent predictor of PIAI. An NRS2002 score ≥ 3 signals malnutrition or nutritional risk in patients. Malnutrition reduces immune cell numbers, and also inhibits their activation and functionality. As a result, the body’s pathogen-defense weakens, significantly raising the risk of PIAI. Many studies have indicated that NRS2002 scores ≥ 3 are often present in patients with gastric or colorectal cancer (17, 32, 33). Furthermore, according to the Global Leadership Initiative on Malnutrition (GLIM) criteria, the initial step involves identifying patients at risk of malnutrition through validated screening tools (34). In this study, the NRS2002 served this purpose. The second step of the GLIM criteria is to diagnose and evaluate malnutrition severity, which requires at least one phenotypic indicator and at least one etiological indicator (34). Our previous study found that GLIM-defined malnutrition is an independent risk factor for serious postoperative complications in gastric cancer patients (35). Therefore, it is necessary to strengthen nutritional risk screening and improve the diagnosis rate of malnutrition in order to implement timely and effective nutritional treatment and improve patient prognosis.

The results of this study showed that elevated CRP levels on POD 1 and POD 3 were independent risk factors for PIAI after gastrointestinal surgery, which was consistent with several studies (36–38). However, some studies have revealed that due to the low positive predictive value of CRP, relying merely on CRP lacks the capacity to diagnose PIAI, as it has poor sensitivity in predicting PIAI in some cases (10, 13, 39). This is because postoperative CRP elevation may be caused by physiological fluctuations resulting from preoperative bowel preparation or transient bacterial contamination during surgery. To address this, our study combined CRP with other biomarkers to enhance the predictive accuracy. By integrating multiple biomarkers, we aim to utilize the unique strengths of each marker while compensating for their individual limitations, with the goal of enhancing the overall performance of our predictive models. In our study, the AUC values of 0.731 and 0.834 in the development cohort, and 0.958 and 0.951 in the validation cohort, demonstrating the effectiveness of this approach.

Both PCT and IL-6 have been confirmed to be associated with infectious diseases, especially abdominal infections (40). In this study, only IL-6 was identified as a predictor of PIAI on POD 1, which concurs with the results of other studies (41, 42). A meta-analysis has indicated that in patients undergoing gastrointestinal surgery, PCT is only moderately effective as a diagnostic test for postoperative infection or sepsis, while IL-6 exhibits superior performance, with a sensitivity of 0.84 (40). While both have limited specificity in colorectal resection surgery, they perform better in upper gastrointestinal surgery (40). Moreover, in clinical practice, compared to CRP, PCT is 25-fold costly yet offers no significant diagnostic advantages (43). This cost-effectiveness imbalance prompts clinicians to consider diagnostic accuracy and economic factors when choosing detection indicators. With its good predictive performance for postoperative infections and a relatively reasonable cost-effectiveness, IL-6 may deserve attention in clinical settings, especially in the early warning of infections related to gastrointestinal surgery.

Previous studies have revealed that DF indicators can predict postoperative pancreatic and biliary fistulas and have explored their links to abdominal infections after gastrointestinal surgery (15, 44, 45). However, most of these studies have focused primarily on identifying relevant risk factors. In our study, we integrated serological and DF indicators to construct two predictive models based on the identification of independent risk factors. Specifically, regarding the DF indicators, we found that LDH and TP in the DF were strongly associated with PIAI on POD 1, while LDH remained a strong correlation with PIAI on POD 3. LDH is a widely distributed enzyme in cells and when tissue damage or inflammation occurs, LDH may be released into the DF, making it a potential biomarker for local inflammatory changes (46, 47). This finding was consistent with a previous study suggesting that LDH in DF could serve as a biomarker for postoperative complications and anastomotic leak in colorectal patient (15). In addition to LDH, TP has been suggested to reflect inflammatory processes and tissue integrity (48). Elevated TP levels in DF may indicate increased protein leakage due to tissue damage or an ongoing inflammatory process, which could be potentially related to the development of PIAI. These DF indicators are readily measurable and can provide early insights into local inflammatory changes, making them valuable complements to traditional serological markers. In contrast, a single-center prospective cohort study has identified that DF amylase could serve as a biomarker for detecting anastomotic leak after rectal resection (49). However, our retrospective study found no significant correlation between DF amylase and PIAI. This discrepancy may be attributed to the broader patient population and surgical procedures included in our study, which could have obscured the association. Additionally, inconsistent sampling in our retrospective design may have missed critical time points for detecting transient elevations in DF amylase levels, unlike the predefined and consistent sampling schedules in prospective studies. Furthermore, although ascites glucose was not identified as a predictive factor for PIAI in our study, we postulate it could be a potential influencing factor. Previous studies have indicated that during abdominal infections, glucose in DF tends to decrease because of bacterial consumption of ascitic sugar (50, 51). The retrospective nature of this study limited our ability to collect concurrent venous blood glucose levels when collecting ascites. This limitation may have hindered our ability to fully assess the role of glucose in PIAI development, highlighting the need for further research to verify its potential influence.

To enhance clinical effectiveness, we hope to integrate nomogram use into hospital information systems and train healthcare professionals accordingly. The clinical workflow involves collecting required indicators on POD 1 and POD 3, calculating the patient’s predicted PIAI probability using the nomogram, and initiating early intervention if the predicted probability exceeds the cutoff value. For patients with an NRS2002 score ≥ 3, prompt nutritional support should be initiated (52). High-risk patients should undergo enhanced monitoring, including frequent testing of infection markers. Prophylactic antibiotics may be considered, especially with clinical signs of infection. A multidisciplinary team (MDT) comprising surgeons, infectious disease specialists, nutritionists, and nursing staff should comprehensively manage these high-risk patients.

This study has several limitations. Firstly, due to variations in the start time of surgery, the interval between surgery completion and the collection of blood samples on POD1 may vary among patients. Considering the dynamic changes in the levels of early inflammatory markers (CRP, CPT, IL-6), which may cause fluctuations in the levels of infection markers regardless of postoperative complications, it would be more suitable for clinical practice to have individualized and precise sampling times for each patient. Secondly, as a two-center retrospective study, it failed to prospectively collect some indicators, such as concurrent venous blood glucose with DF collection. Additionally, daily dynamic monitoring of serum and ascites DF infection indicators was lacking, which would have provided more comprehensive trend-tracking. Future studies should address these limitations through prospective designs with standardized sampling protocols and comprehensive biomarker profiling.

## 5 Conclusions

This study established two predictive models for postoperative abdominal infections in gastrointestinal tumors patients on POD 1 and POD 3, based on NRS2002, serum infection markers, and abdominal DF indicators. These models are simple and feasible, with good discrimination and calibration capabilities. We believe that they could enable clinicians to promptly identify patients at high risk for postoperative infections, facilitating early preventive interventions and enhancing patient outcomes.

## Data Availability

The raw data supporting the conclusions of this article will be made available by the authors, without undue reservation.

## References

[1] Dekker E Tanis PJ Vleugels JLA Kasi PM Wallace MB. Colorectal cancer. Lancet. (2019) 394:1467–80. doi: 10.1016/s0140-6736(19)32319-0 31631858

[2] Smyth EC Nilsson M Grabsch HI van Grieken NC Lordick F. Gastric cancer. Lancet. (2020) 396:635–48. doi: 10.1016/s0140-6736(20)31288-5 32861308

[3] Yu Z Liang C Xu Q Li R Gao J Gao Y. Analysis of postoperative complications and long term survival following radical gastrectomy for patients with gastric cancer. Sci Rep. (2024) 14:23869. doi: 10.1038/s41598-024-74758-x 39396097 PMC11470947

[4] Shimada H Fukagawa T Haga Y Oba K. Does postoperative morbidity worsen the oncological outcome after radical surgery for gastrointestinal cancers? A systematic review of the literature. Ann Gastroenterol Surg. (2017) 1:11–23. doi: 10.1002/ags3.12002 29863169 PMC5881350

[5] Alqarni A Kantor E Grall N Tanaka S Zappella N Godement M. Clinical characteristics and prognosis of bacteraemia during postoperative intra-abdominal infections. Crit Care (London England). (2018) 22:175. doi: 10.1186/s13054-018-2099-5 PMC6035454 29980218

[6] Sripathi S Khan MI Patel N Meda RT Nuguru SP Rachakonda S. Factors contributing to anastomotic leakage following colorectal surgery: why, when, and who leaks? Cureus. (2022) 14:e29964. doi: 10.7759/cureus.29964 36381751 PMC9635981

[7] Rennie O Sharma M Helwa N. Colorectal anastomotic leakage: A narrative review of definitions, grading systems, and consequences of leaks. Front Surg. (2024) 11:1371567. doi: 10.3389/fsurg.2024.1371567 38756356 PMC11097957

[8] Bassetti M Eckmann C Giacobbe DR Sartelli M Montravers P. Post-operative abdominal infections: epidemiology, operational definitions, and outcomes. Intensive Care Med. (2020) 46:163–72. doi: 10.1007/s00134-019-05841-5 31701205

[9] Song J Lu Y. Composite inflammatory indicators as early predictor of intra-abdominal infections after general surgery. J Inflammation Res. (2021) 14:7173–9. doi: 10.2147/jir.s340745 PMC8710522 34992412

[10] Tatsuoka T Okuyama T Takeshita E Oi H Noro T Mitsui T. Early detection of infectious complications using C-reactive protein and the procalcitonin levels after laparoscopic colorectal resection: A prospective cohort study. Surg Today. (2021) 51:397–403. doi: 10.1007/s00595-020-02111-6 32785845 PMC7892676

[11] Ljungqvist O Scott M Fearon KC. Enhanced recovery after surgery: A review. JAMA Surg. (2017) 152:292–8. doi: 10.1001/jamasurg.2016.4952 28097305

[12] Rollins KE Lobo DN Joshi GP. Enhanced recovery after surgery: current status and future progress. Best Pract Res Clin Anaesthesiol. (2021) 35:479–89. doi: 10.1016/j.bpa.2020.10.001 34801211

[13] Das U Anandhi A Sureshkumar S Sastry AS Subitha L. Procalcitonin and C-reactive protein as an early predictor of infection in elective gastrointestinal cancer surgery-a prospective observational study. J Gastrointestinal Cancer. (2022) 53:605–13. doi: 10.1007/s12029-021-00661-7 34328613

[14] Wierdak M Pisarska M Kuśnierz-Cabala B Witowski J Dworak J Major P. Changes in plasma albumin levels in early detection of infectious complications after laparoscopic colorectal cancer surgery with eras protocol. Surg Endoscopy. (2018) 32:3225–33. doi: 10.1007/s00464-018-6040-4 PMC5988762 29340818

[15] Agnello L Buscemi S Di Buono G Vidali M Lo Sasso B Agrusa A. Drainage fluid ldh and neutrophil to lymphocyte ratio as biomarkers for early detecting anastomotic leakage in patients undergoing colorectal surgery. Clin Chem Lab Med. (2024) 62:967–78. doi: 10.1515/cclm-2023-1164 37988156

[16] Horan TC Andrus M Dudeck MA. Cdc/nhsn surveillance definition of health care-associated infection and criteria for specific types of infections in the acute care setting. Am J Infection Control. (2008) 36:309–32. doi: 10.1016/j.ajic.2008.03.002 18538699

[17] Kondrup J Rasmussen HH Hamberg O Stanga Z. Nutritional risk screening (Nrs 2002): A new method based on an analysis of controlled clinical trials. Clin Nutr (Edinburgh Scotland). (2003) 22:321–36. doi: 10.1016/s0261-5614(02)00214-5 12765673

[18] Owens WD Felts JA Spitznagel EL Jr. Asa physical status classifications: A study of consistency of ratings. Anesthesiology. (1978) 49:239–43. doi: 10.1097/00000542-197810000-00003 697077

[19] Ajani JA D’Amico TA Bentrem DJ Chao J Cooke D Corvera C. Gastric cancer, version 2.2022, nccn clinical practice guidelines in oncology. J Natl Compr Cancer Network: JNCCN. (2022) 20:167–92. doi: 10.6004/jnccn.2022.0008 35130500

[20] Garg T Chen LY Kim PH Zhao PT Herr HW Donat SM. Preoperative serum albumin is associated with mortality and complications after radical cystectomy. BJU Int. (2014) 113:918–23. doi: 10.1111/bju.12405 PMC4203702 24053616

[21] Liu Y Li L Hu H Yang J Zhang X Chen L. Association between preoperative hematocrit and postoperative 30-day mortality in adult patients with tumor craniotomy. Front Neurol. (2023) 14:1059401. doi: 10.3389/fneur.2023.1059401 36895901 PMC9990837

[22] Zhong B Lin ZY Ma DD Shang ZH Shen YB Zhang T. A preoperative prediction model based on lymphocyte-C-reactive protein ratio predicts postoperative anastomotic leakage in patients with colorectal carcinoma: A retrospective study. BMC Surg. (2022) 22:283. doi: 10.1186/s12893-022-01734-5 35870933 PMC9308913

[23] Li S Li M Wu J Li Y Han J Song Y. Developing and validating a clinlabomics-based machine-learning model for early detection of retinal detachment in patients with high myopia. J Transl Med. (2024) 22:405. doi: 10.1186/s12967-024-05131-9 38689321 PMC11061938

[24] He M Yang L Jia S Yang J Wen X Fan J. Does vitreous haemorrhage and calcification lead to increased risk of enucleation in advanced retinoblastoma? Acta Ophthalmol. (2024) 102:e296–301. doi: 10.1111/aos.15735 37431955

[25] Liu H Dong A Rasteh AM Wang P Weng J. Identification of the novel exhausted T cell cd8+ Markers in breast cancer. Sci Rep. (2024) 14:19142. doi: 10.1038/s41598-024-70184-1 39160211 PMC11333736

[26] SĂĄnchez-VelĂĄzquez P Pera M JimĂŠnez-Toscano M Mayol X RogĂŠs X Lorente L. Postoperative intra-abdominal infection is an independent prognostic factor of disease-free survival and disease-specific survival in patients with stage ii colon cancer. Clin Transl Oncol. (2018) 20:1321–8. doi: 10.1007/s12094-018-1866-8 29623587

[27] Zhu R Hong X Zhang D Xiao Y Xu Q Wu B. Application of metagenomic sequencing of drainage fluid in rapid and accurate diagnosis of postoperative intra-abdominal infection: A diagnostic study. Int J Surg. (2023) 109:2624–30. doi: 10.1097/js9.0000000000000500 PMC10498887 37288562

[28] Facy O Paquette B Orry D Binquet C Masson D Bouvier A. Diagnostic accuracy of inflammatory markers as early predictors of infection after elective colorectal surgery: results from the imacors study. Ann Surg. (2016) 263:961–6. doi: 10.1097/sla.0000000000001303 26135691

[29] Domínguez-Comesaña E Estevez-Fernández SM López-Gómez V Ballinas-Miranda J Domínguez-Fernández R. Procalcitonin and C-reactive protein as early markers of postoperative intra-abdominal infection in patients operated on colorectal cancer. Int J Colorectal Dis. (2017) 32:1771–4. doi: 10.1007/s00384-017-2902-9 28918433

[30] Akimoto E Kinoshita T Sato R Yura M Harada J Yoshida M. Impact of postoperative intra-abdominal infectious complications on survival outcomes in patients with gastric cancer who underwent laparoscopic surgery. Surg Endoscopy. (2023) 37:382–90. doi: 10.1007/s00464-022-09522-1 35969298

[31] Fujiya K Kumamaru H Fujiwara Y Miyata H Tsuburaya A Kodera Y. Preoperative risk factors for postoperative intra-abdominal infectious complication after gastrectomy for gastric cancer using a Japanese web-based nationwide database. Gastric Cancer: Off J Int Gastric Cancer Assoc Japanese Gastric Cancer Assoc. (2021) 24:205–13. doi: 10.1007/s10120-020-01083-3 32440807

[32] Ruan X Wang X Zhang Q Nakyeyune R Shao Y Shen Y. The performance of three nutritional tools varied in colorectal cancer patients: A retrospective analysis. J Clin Epidemiol. (2022) 149:12–22. doi: 10.1016/j.jclinepi.2022.04.026 35537604

[33] Li YF Nie RC Wu T Li SM Chen S Wang W. Prognostic value of the nutritional risk screening 2002 scale in metastatic gastric cancer: A large-scale cohort study. J Cancer. (2019) 10:112–9. doi: 10.7150/jca.27729 PMC6329866 30662531

[34] Cederholm T Jensen GL Correia M Gonzalez MC Fukushima R Higashiguchi T. Glim criteria for the diagnosis of malnutrition - a consensus report from the global clinical nutrition community. J Cachexia Sarcopenia Muscle. (2019) 10:207–17. doi: 10.1002/jcsm.12383 PMC6438340 30920778

[35] Sun S Huang W Wang Z Xie W Zhou J He Q. Association of malnutrition diagnosed using global leadership initiative on malnutrition criteria with severe postoperative complications after gastrectomy in patients with gastric cancer. J Laparoendoscopic Advanced Surg Techniques Part A. (2023) 33:1193–200. doi: 10.1089/lap.2023.0310 37787912

[36] Asti E Bonitta G Melloni M Tornese S Milito P Sironi A. Utility of C-reactive protein as predictive biomarker of anastomotic leak after minimally invasive esophagectomy. Langenbeck’s Arch Surg. (2018) 403:235–44. doi: 10.1007/s00423-018-1663-4 29516256

[37] Benoit O Faron M Margot N Creavin B Debove C Tiret E. C-reactive protein values after colorectal resection: can we discharge a patient with a C-reactive protein value >100? A retrospective cohort study. Dis Colon Rectum. (2019) 62:88–96. doi: 10.1097/dcr.0000000000001216 30451748

[38] Shi J Wu Z Wang Q Zhang Y Shan F Hou S. Clinical predictive efficacy of C-reactive protein for diagnosing infectious complications after gastric surgery. Ther Adv Gastroenterol. (2020) 13:1756284820936542. doi: 10.1177/1756284820936542 PMC7339084 32670413

[39] de Mooij CM Maassen van den Brink M Merry A Tweed T Stoot J. Systematic review of the role of biomarkers in predicting anastomotic leakage following gastroesophageal cancer surgery. J Clin Med. (2019) 8:2005. doi: 10.3390/jcm8112005 31744186 PMC6912692

[40] Jerome E McPhail MJ Menon K. Diagnostic accuracy of procalcitonin and interleukin-6 for postoperative infection in major gastrointestinal surgery: A systematic review and meta-analysis. Ann R Coll Surgeons Engl. (2022) 104:561–70. doi: 10.1308/rcsann.2022.0053 PMC9433179 36044921

[41] Procházka V Lacina L Smetana K Jr. Svoboda M Skřivanová K Beňovská M. Serum concentrations of proinflammatory biomarker interleukin-6 (Il-6) as a predictor of postoperative complications after elective colorectal surgery. World J Surg Oncol. (2023) 21:384. doi: 10.1186/s12957-023-03270-9 38098074 PMC10720211

[42] Zhao G Zhu J Shi C Wang D Wu W Kuang T. Serum interleukin-6 as a biomarker for early prediction of post-operative infectious complications after elective pancreatectomy. Surg Infect. (2023) 24:811–7. doi: 10.1089/sur.2023.108 37906123

[43] Cousin F Ortega-Deballon P Bourredjem A Doussot A Giaccaglia V Fournel I. Diagnostic accuracy of procalcitonin and C-reactive protein for the early diagnosis of intra-abdominal infection after elective colorectal surgery: A meta-analysis. Ann Surg. (2016) 264:252–6. doi: 10.1097/sla.0000000000001545 27049766

[44] Jin S Shi XJ Wang SY Zhang P Lv GY Du XH. Drainage fluid and serum amylase levels accurately predict development of postoperative pancreatic fistula. World J Gastroenterol. (2017) 23:6357–64. doi: 10.3748/wjg.v23.i34.6357 PMC5603503 28974903

[45] Sugawara T Shindoh J Nishioka Y Hashimoto M. Total bilirubin amount in drainage fluid can be an early predictor for severe biliary fistula after hepatobiliary surgery. Biosci Trends. (2017) 11:588–94. doi: 10.5582/bst.2017.01208 29070759

[46] Manosalva C Quiroga J Hidalgo AI AlarcĂłn P Anseoleaga N Hidalgo MA. Role of lactate in inflammatory processes: friend or foe. Front Immunol. (2021) 12:808799. doi: 10.3389/fimmu.2021.808799 35095895 PMC8795514

[47] Fang Y Li Z Yang L Li W Wang Y Kong Z. Emerging roles of lactate in acute and chronic inflammation. Cell Commun Signal. (2024) 22:276. doi: 10.1186/s12964-024-01624-8 38755659 PMC11097486

[48] Zeng RX Xu JP Zhang YZ Tan JW Kong YJ Zhang MZ. Associations of total protein, albumin, and globulin with insulin resistance: an nhanes study. Front Endocrinol (Lausanne). (2024) 15:1393137. doi: 10.3389/fendo.2024.1393137 39345890 PMC11427264

[49] Clark DA Edmundson A Steffens D Harris C Stevenson A Solomon M. Drain Fluid Amylase as a Biomarker for the Detection of Anastomotic Leakage after Rectal Resection without a Diverting Ileostomy. ANZ J Surg. (2022) 92:813–8. doi: 10.1111/ans.17461 34994080

[50] Mao JY Li DK Zhang D Yang QW Long Y Cui N. Utility of paired plasma and drainage fluid mngs in diagnosing acute intra-abdominal infections with sepsis. BMC Infect Dis. (2024) 24:409. doi: 10.1186/s12879-024-09320-1 38632536 PMC11022345

[51] Zhou Q He W Liu Y Liao B Liang Y Mo B. Drainage volume on postoperative day one to predict clinically relevant postoperative pancreatic fistula following distal pancreatectomy. BMC Surg. (2022) 22:297. doi: 10.1186/s12893-022-01748-z 35909183 PMC9341036

[52] Hersberger L Bargetzi L Bargetzi A Tribolet P Fehr R Baechli V. Nutritional risk screening (Nrs 2002) is a strong and modifiable predictor risk score for short-term and long-term clinical outcomes: secondary analysis of a prospective randomised trial. Clin Nutr. (2020) 39:2720–9. doi: 10.1016/j.clnu.2019.11.041 31882232

